# Novel insights into the behavioral analysis of mice subjected to the forced-swim test

**DOI:** 10.1038/tp.2015.44

**Published:** 2015-04-14

**Authors:** L Chen, G C Faas, I Ferando, I Mody

**Affiliations:** 1 Molecular, Cellular and Integrative Physiology Interdepartmental Ph.D. Program, University of California Los Angeles, Los Angeles, CA, USA; 2 Department of Neurology, The David Geffen School of Medicine at UCLA, University of California Los Angeles, Los Angeles, CA, USA; 3 Department of Physiology, The David Geffen School of Medicine at UCLA, University of California Los Angeles, Los Angeles, CA, USA

## Abstract

The forced-swim test (FST) is one of the most widely used rodent behavioral assays, in which the immobility of animals is used to assess the effectiveness of antidepressant drugs. However, the existing, and mostly arbitrary, criteria used for quantification could lead to biased results. Here we believe we uncovered new confounding factors, revealed new indices to interpret the behavior of mice and propose an unbiased means for quantification of the FST.

## Introduction

Major depressive disorder is a key public health concern. The results of the first Global Burden of Disease study in 1990 revealed a group of disorders that primarily cause nonfatal burden, quantified by years lived with disability and disability-adjusted life years.^1^ According to data from the Global Burden of Disease study 2010,^2, 3, 4^ mental and substance disorders accounted for 7.4% of disability-adjusted life years, and they were the leading cause (22.9%) of years lived with disability worldwide.^4^ Within the mental and substance disorders group, major depressive disorder contributed most of the disability-adjusted life years and years lived with disability, with a global point prevalence of 4.7%.^5^ From 1990 to 2010, the contribution of major depressive disorder increased by 37% to both disability-adjusted life years^3^ and years lived with disability.^2^ Also, major depressive disorder was predicted to be the second leading contributor to the burden of diseases by 2020.^6^

Clinically, depression is a heterogeneous disorder in humans, and it is difficult to model the psychological aspect of the disease in animals.^7^ There are just a limited number of tests available for detecting a ‘depressed' phenotype in rodents;^7, 8, 9^ among those, the forced-swim test (FST) is one of the most widely used tests across laboratories for assessing symptoms of depression. FST was originally introduced by Porsolt *et al.* in rats^10, 11^ and mice^12^ for the screening of antidepressants. In this test, if treatment with a drug reduced behavioral immobility,^10, 12, 13^ thought of as a measure of despair, the drug could be considered as an ‘antidepressant'.^10, 11, 12^ The advantages of the FST consist of its ease of use, reliability across different laboratories and trials and the ability to detect a broad spectrum of antidepressants.^14, 15^ However, the test also has some drawbacks, such as its unreliability in the detection of the effects of selective 5-HT reuptake inhibitors (selective serotonin reuptake inhibitors), which is a major group of antidepressant drugs.^13^ Another important question is how to interpret the immobility of the animals in the FST. Researchers have been arguing that immobility is largely dependent on learning and memory in the rat FST,^14, 16^ but only limited research has been done on the role of cognitive processes in the mouse FST. As the FST was originally developed for rats, a species much better adapted to water than mice, some technical issues remain related to using mice in FST, a species for which the test remains to be adequately adapted.^7, 8^ In Porsolt's original FST for rats, the animals will go through two exposures.^10, 11^ The first exposure was to induce a stable level of immobility, and the second exposure was to quantify the immobility after drug or vehicle treatment, whereas for reasons yet unclear, mice show a sufficiently stable level of immobility during the last 4 min of a 6-min swim test.^13^ Also, particularly in mice, the immobility may be influenced by factors other than a variation in emotional state,^17^ which renders the interpretation of the results dependent on the strain of mice,^18, 19, 20^ the water temperature^21, 22^ or possibly other factors. Despite the disadvantages inherent to carrying out FST in mice and the inability to really measure depression, the test is still widely used as some quantification of depressive behavior. The ability and potential to modify mice genetically, thus enabling better insights into molecular mechanisms underlying mental disorders, has created a demand for better adapting the test to this rodent species. To date, almost 40 strains of mice have been generated with a depression or antidepressant-related phenotype.^7^

Over the years, researchers have made modifications to the FST to enhance its sensitivity, specificity and reliability.^15, 17, 23, 24, 25^ In this study, we set out to determine whether it is possible to quantify some of the factors affecting the behavior of mice. First, we revealed buoyancy as a confounding factor in the FST. We also devised new unbiased quantitative measurements of the behavior of mice, including developing a systematic way to determine the latency to immobility, and discovering an oscillatory pattern of behaving mice in the FST.

## Materials and methods

### Animals

All animal use was approved by the UCLA Chancellor's Animal Research Committee. All mice used for the FST were on C57BL/6J background and were obtained directly from Jackson Labs or bred in the UCLA animal facilities. Mice were kept in the vivarium on a 12-h light/dark cycle with free access to water and food. Calbindin knockout (*Calb1*^−^^*/−*^) mice weighing between 32 and 43 g (male, 62–70 weeks old) were used for the customized 8-min FSTs (see below). Alpha5 subunit containing GABA_A_ receptor knockout heterozygous (*Gabra5*^*+/−*^) mice (male and female, 10–13 weeks old) were used for some of the standard 6-min FSTs. For all other FSTs, wild-type (WT) mice weighing between 20 and 25 g (male, 10–18 weeks old) were used.

### Standard 6-min FST

**Figure 1:**
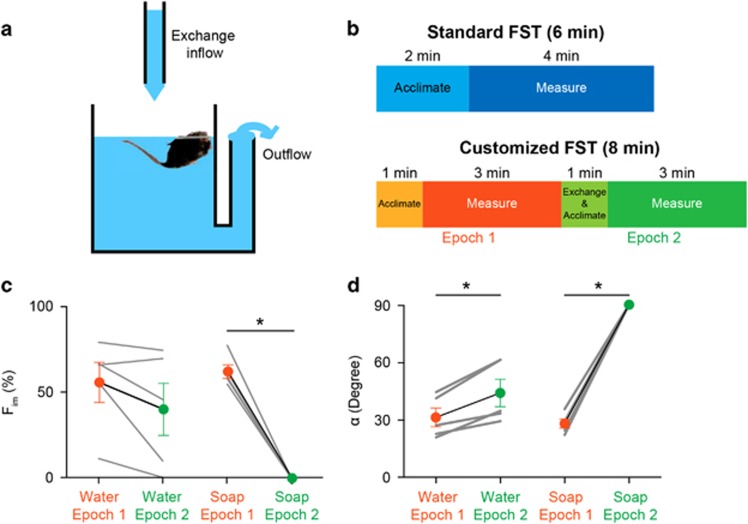
Customized setup for liquid exchange in the FST. (**a**) The diagram of the customized setup, showing the exchange inflow of the new liquid and the outflow of the original liquid in the container. (**b**) Two experiment protocols for the standard FST (6 min) and customized FST (8 min). (**c** and **d**) Exchanging water for 1 l of water (*n*=5) or 0.5% soap solution (*n*=5) using the customized setup. Fraction of total immobile time (*F*_im_) (**c**) and angles (*α*) (**d**) of the animals both changed significantly after exchanging water for soap solution, going from epoch 1 (orange) to epoch 2 (green). The gray lines show the changes in individual mice from epoch 1 to epoch 2, and the colored filled circles show the averaged data in each epoch. FST, forced-swim test.

All 6-min FSTs were carried out using C57BL/6J mice (male and female, 8–70 weeks old, 20–43 g, see Animals section). The animals swam in standard 2 l glass beakers (diameter 13.1 cm) filled with water to 4.5 cm from the top so that the animals could neither touch the bottom with their tails, nor escape from the top. Before each test, the container was thoroughly cleaned. Water temperature was kept between 23 °C and 26 °C.^10, 11, 12, 17^ At the start of each test, an animal was gently picked up by its tail from the home cage and rapidly placed into the middle of the container. At the time the animal was placed in the water, the recording time was started and the duration of each standard FST was set to 6 min (for a customized FST, see below). The entire FST session was videotaped for later analysis (Figure 1b). After the test, the animal was removed from the water, dried with a towel and put into a warm cage (temperature of bedding 31–33 °C) for 15 min before returning to their home cage. All animals were first-time swimmers and none were used for multiple FSTs.

### Customized 8-min FST

For the customized FST, the setup for the FST was adapted for liquid exchange (Figure 1a). The container was made of transparent plexiglass. It was designed to be large enough (diameter 14.6 cm, height 27.9 cm) so that the animals could neither touch the bottom with their tails, nor escape from the top. To keep the level of water constant in the container during a liquid exchange, a thick (diameter 2.6 cm) vertical plastic pipe was connected to the container at its side, making a communicating vessel structure with the container, so that any excess liquid above 21.0 cm from the bottom could flow out. A 1.0-cm diameter plastic inflow pipe was fixed above the container to allow the exchange of solutions. The inflow was positioned above the center of the container so that the incoming liquid would not disturb the swimming animals, as mice swim along the edge of the container. In this manner, the entire process of exchanging all of the 3 l capacity of the container could be done within 30 s (Figure 1b). In contrast to the 6-min FST with 2 min of acclimatization and 4 min for quantification, the 8-min FST had two swim epochs. In epoch 1, mice swam in water for 4 min. After this, epoch 2 started with a liquid exchange lasting <30 s. After a total duration of 4 min for epoch 2, the animals were removed form the water and dried with a towel and put into a warm cage (temperature of bedding 31–33 °C) for 15 min before returning to their home cage. All the animals were first-time swimmers and none were used for multiple FSTs.

### Analysis

#### (1) Definition of immobility

All FST videos were scored by one individual in a double-mask manner. The scores were assigned as ‘0' for immobility and ‘1' for mobility with a time resolution of 0.1 s. A mouse was considered immobile when floating and/or making only necessary movements to keep the balance of its body or to keep its head above the water.^10, 11, 12, 13^ According to the current standard of general FST analyses, latency to immobility (*t*_lat_) was defined as the time from start to the first bout of immobility lasting longer than 1 s,^13^ unless otherwise stated. Fractions of total immobile time (*F*_im_) were calculated as the total immobile time divided by the total swimming time used to calculate the immobility.

#### (2) Scoring of the FSTs

For the standard FST (6 min), the first 2 min were considered a time for the animals to explore and acclimate to the environment. For the full 6 min, mobility/immobility were scored, but for the calculation of *F*_im_, only the data of the last 4 min were used (*F*_im_=total immobile time during the last 4 min/240 s). For the calculations involving the calculation of *t*_lat_, data of all 6 min were used.

For the customized FST (8 min), the first 1 min at the start of each epoch was considered a time for the animals to explore and acclimate to the environment. In each epoch, the full 4 min mobility/immobility were scored, but for the calculation of *F*_im,_ only the data of the last 3 min were used (*F*_im_=total immobile time during the last 3 min/180 s). For the calculations involving the calculation of *t*_lat_, data of all 4 min in each epoch were used.

#### (3) Angle during immobility

**Figure 2:**
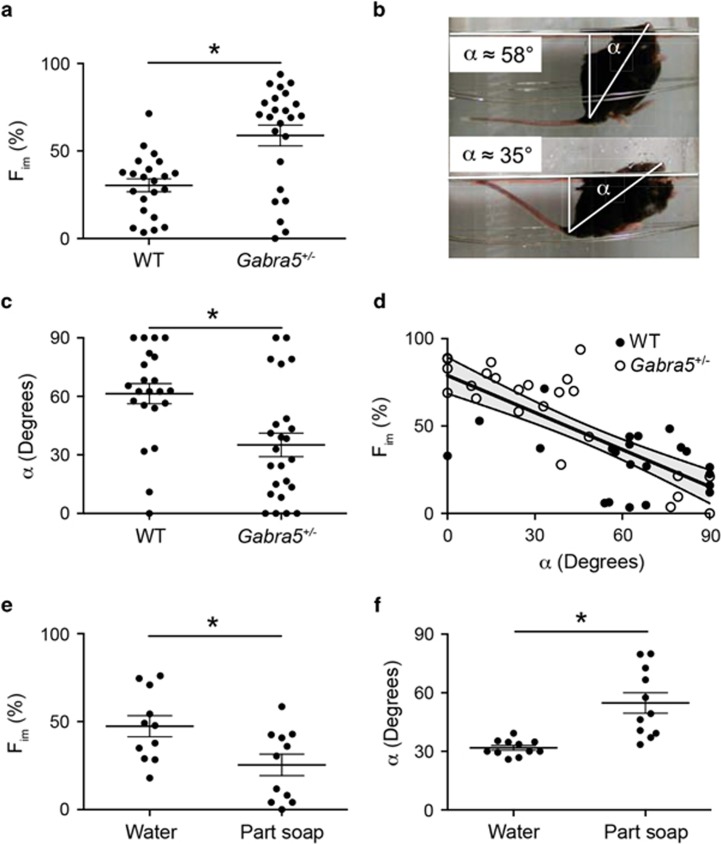
Strong inverse correlation between body posture angle in water and immobility. (**a**) Significant difference in the fractions of total immobile time (*F*_im_) of WT (*n*=22) and *Gabra5*^*+/−*^ (*n*=24) mice. (**b**) Two immobile mice with different angles (*α*) in water. White lines indicate how the angles were measured. Pictures of mice were taken in experiments shown in **e** and **f**. (**c**) Significant difference in *α* of WT and *Gabra5*^*+/−*^ mice. (**d**) *F*_im_ plotted as a function of *α* shows a strong inverse correlation between *α* and *F*_im_. (**e** and **f**) Significant difference in *F*_im_ (**e**) and *α* (**f**) of mice with water (Water, *n*=11) or 1% soap solution (Part soap, *n*=11) applied to their caudal areas. WT, wild-type.

We defined the angle (*α*) of an immobile mouse with respect to the water surface. After the acclimatization period (2 min for standard FST, 1 min for each epoch of the customized FST), one frame was taken out from each video at the first moment when the mouse stopped swimming and was in full profile view. The angle *α* was determined using the depth of the base of the tail and the distance between the intersection of the body with the water and the base of the tail (white lines, Figure 2b).

#### (4) Defining new thresholds for screening the first critical immobility

The durations of bouts of immobility (*t*_im_) were taken from the entire swim session of each mouse. The cumulative probability of the immobility durations (*Φ*(*t*_im_)) was fitted with two cumulative normal distributions with the following equation:


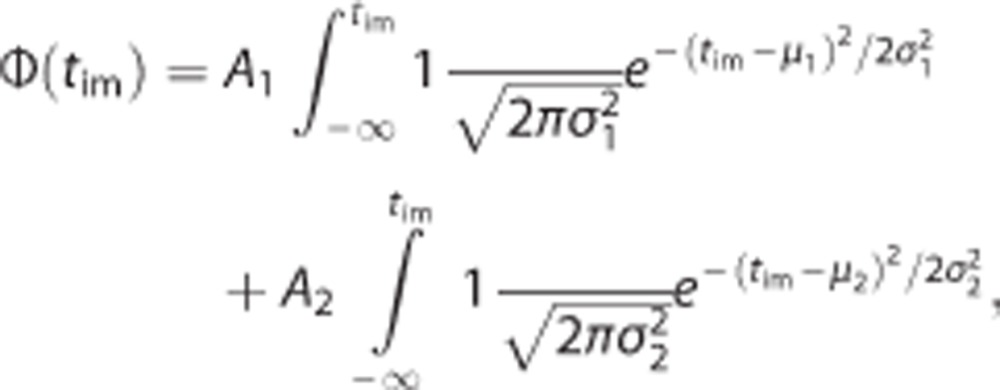


where *A*_1_+*A*_2_=1.

In the equation, *t*_im_ is the duration of each bout of immobility; *A, μ* and *σ* are the amplitudes, means and standard deviations, respectively, of the two normal distributions. The distributions were fitted using the NORM.DIST function in Microsoft Office Excel 2011.

To make sure that the *Φ*(*t*_im_) of *t*_im_ can be fitted with two rather than one normal distribution, we did a F-test to validate whether two normal distributions fit the data significantly better than a single distribution. For the F-test, we fitted the *Φ*(*t*_im_) curves with both one and two normal distributions. When fitting with one distribution, *A*_1_=1 and *A*_2_=0 in the equation shown above. The *F*-values were calculated with the following equation:


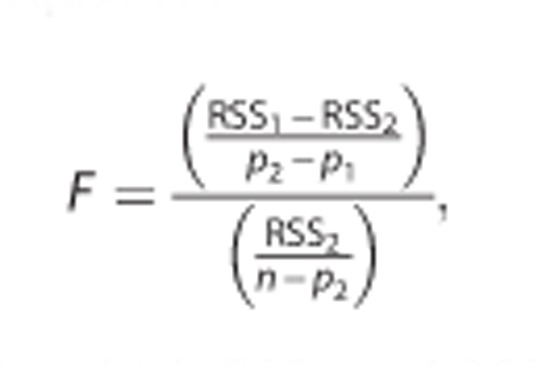


in which RSS_1_ and RSS_2_ are the residual sums of squares from fitting with one and two normal distributions, respectively; *p*_1_ and *p*_2_ are the number of parameters used for fitting with one and two normal distributions, respectively; and *n* is the number of points used for fitting. According to the equation of the normal distribution, *p*_1_=2 and *p*_2_=5.

**Figure 3:**
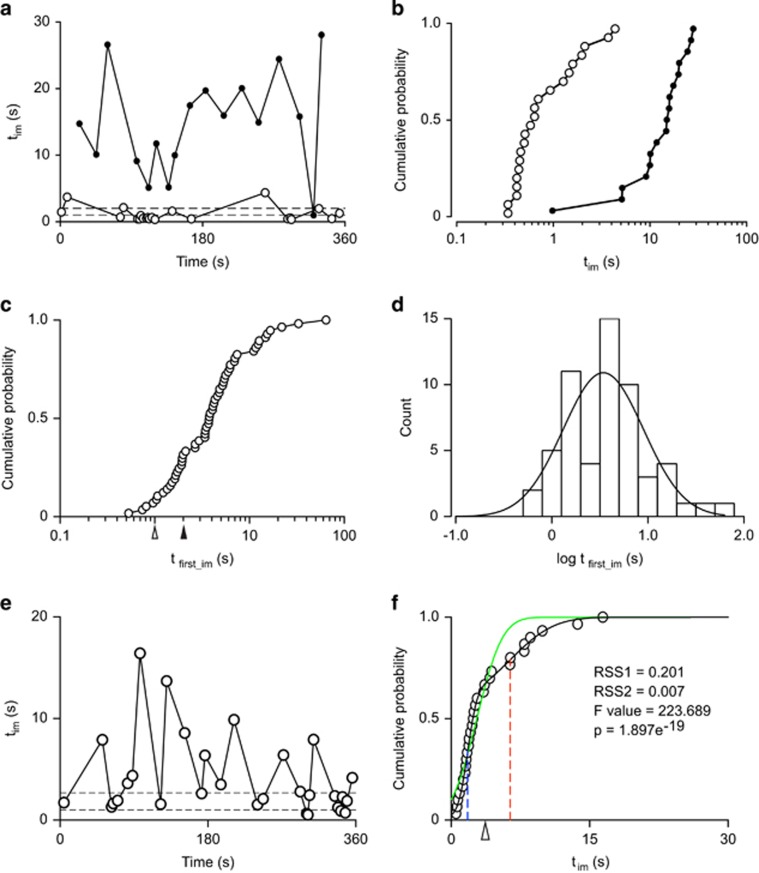
The distribution of the durations of bouts of immobility (*t*_im_). (**a**) Examples of one mouse with longer *t*_im_s (filled circles) and the other with shorter *t*_im_s (open circles) during the entire swim session of 6 min. The *t*_im_s are plotted against the time at which the immobility occurred. The two dashed lines show the traditional arbitrary thresholds of 1 s and 2 s for screening the first bout of immobility. (**b**) Cumulative probability of the *t*_im_s of the two mice shown in **a**. (**c**) Cumulative probability of the *t*_first_im_s of 57 mice used in the 6-min FST. The open and filled triangles show the traditional arbitrary thresholds of 1 s and 2 s, respectively, for the calculation of *t*_lat_. (**d**) The histogram of the log values of all the *t*_first_im_s shown in **c**. The histogram follows a log-normal distribution. (**e**) Immobility durations of a mouse (*t*_im_) plotted against the time at which the immobility occurred during the 6-min FST. The two dashed lines show the traditional arbitrary thresholds of 1 s and 2 s for screening the first bout of immobility. (**f**) Cumulative probability of the *t*_im_s shown in **a**. The open circles are fitted with either two normal distributions (black line) or one normal distribution (green line). The residual sum of squares (RSS) when fitting with one or two distributions are RSS1=0.201 and RSS2=0.007, respectively. The *F*-value calculated with RSS1 and RSS2 is 223.689 (*P*=1.897e^*−*19^). When fitting with two normal distributions, the blue dashed line is the mean of the first distribution, the red dashed line is the mean of the second distribution and the open triangle shows the new threshold (*t*_c_) for screening the first critical immobility calculated as the weighted mean of the two distributions. FST, forced-swim test; *t*_c_, critical threshold.

We then performed the F-test and calculated *P*-values were using the F.DIST function in Microsoft Office Excel 2011. As an example, for the mouse shown in Figure 3f, when we were fitting the *Φ*(*t*_im_) curve with one normal distribution (Figure 3f, green line), the RSS was RSS1=0.201, whereas the RSS decreased to RSS2=0.007 when fitting with two normal distributions (Figure 3f, black line). Accordingly, the *F*-value calculated from RSS1 and RSS2 was 223.689, and the *P*-value we got from the F-test was 1.897e^−19^, which means that two distributions fit the *Φ*(*t*_im_) curve significantly better than one distribution for this mouse.

Of the 68 mice used in our tests, only in 7 mice (~10%) two normal distributions failed to fit the data significantly better than a single normal distribution. Therefore, there are two distinct populations in the distributions of *t*_im_s in 90% of the swim tests.

When fitting *Φ*(*t*_im_) with two normal distributions, the new threshold (*t*_c,_ critical threshold) for screening the first critical immobility was defined as a weighted mean as follows:


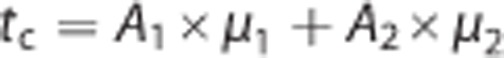


In the equation, *A* and *μ* are the amplitudes and means, respectively, of the two normal distributions. For the seven cases with a single distribution, *t*_c_=*μ*_1_ was used as the threshold value.

The new latency to immobility (*t*_lat_) was then calculated as the latency to the first bout of immobility that was longer than *t*_c_.

#### (5) Oscillations in swimming probability

**Figure 4:**
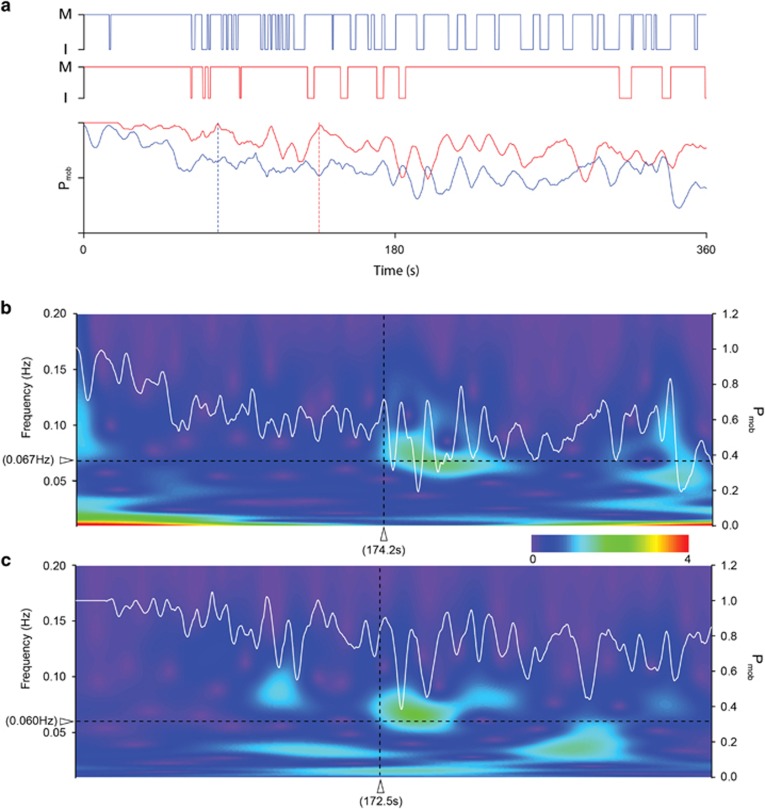
Probability of being mobile (*P*_mob_) plots. (**a**) Probability of being mobile (*P*_mob_) over the entire swim test shown in Figures 2e and f. The top two plots represent the binary states (M for mobile; I for immobile) of two individual mice with water (blue lines, top plot) or 1% soap solution (red lines, middle plot) applied to their caudal areas. The blue and red lines in the bottom plot represent the averages of the behavior, which is *P*_mob_, of 11 mice in each group. The blue and red dashed lines show the averaged *t*_lat_s calculated with *t*_c_s obtained for each mouse in the two groups. (**b** and **c**) Morlet wavelet transform of the *P*_mob_ plots shown in **a**, where color intensity indicates amplitude of a certain frequency component (y axis) as a function of time (x axis) of the *P*_mob_ plots. The rainbow color bar shows the corresponding color scale for the oscillation amplitudes in arbitrary units. A strong ‘oscillation' in the swimming probability (green areas indicating a repeated stop-and-swim behavior with a certain frequency) with a comparable average frequency (0.060 and 0.067 Hz) occurred at similar times (174.2 s and 172.5 s) in the *P*_mob_ plots of mice regardless whether water (**b**) or 1% soap solution (**c**) was applied to their caudal areas. Even if the animals would have similar oscillating behavior, if they were out of phase with each other, the oscillating behavior would not appear in the averages, that is, the *P*_mob_ plots. *t*_c_, critical threshold; *t*_lat_, latency to immobility.

The oscillations were determined using the Morlet wavelet transforms (Figures 4b and c) on the summed probability of being mobile (*P*_mob_) plots (Figure 4a) after smoothing by a fourth order Savitzky–Golay algorithm (Igor 64, WaveMetrics, Lake Oswego, OR, USA).

### Statistics

All the results are expressed as mean±s.e.m. Statistical differences between control groups and experimental groups were determined by unpaired two-tailed Mann–Whitney test unless otherwise stated. *P*<0.05 was considered to be statistically significant.

### Equipment

The behavior of mice during the swim test was recorded using a Casio Exilim camera (Dover, NJ, USA). The software used for scoring was Etholog (Eduardo B. Ottoni, University of São Paulo, São Paulo, Brazil). The Morlet wavelet transform was done in Igor Pro 6 (WaveMetrics). All other data analyses were done in Microsoft Excel for Mac 2011 and Prism 6 (GraphPad, La Jolla, CA, USA). Johnson's baby head-to-toe wash (Johnson & Johnson, Skillman, NJ, USA) was mixed with warm tap water (23–26 °C) to make a mild 0.5–1% soap solution. This soap solution was used in certain swim tests to reduce surface tension of the water, diminishing air trapped in the fur. Consequently the total buoyancy of animals in a soap solution will be decreased.

## Results

For research not specifically described here, WT and α5 subunit containing GABA_A_ receptor heterozygous (*Gabra5*^+/−^) mice were used in a standard 6-min FST (See Materials and Methods). There was a significant difference between the fractions of total immobile time (*F*_im_) of WT and *Gabra5*^+/−^ mice (*F*_im_WT_ =30.4±3.7% vs *F*_im___Gabra5+/−_ =58.9±5.9%, *P*=0.0010, Figure 2a). However, we also observed remarkable differences in the animals' floating postures, which we quantified by measuring the angles formed between the surface of the water and the animals' axis while immobile (*α*, Figure 2b, See Materials and Methods). In WT mice, *α* was significantly larger than in *Gabra5*^+/−^ mice (*α*_WT_=61±5° vs *α*_Gabra5+/−_=35±6°, *P*=0.0028, Figure 2c). Furthermore, *α* inversely correlated with *F*_im_ (*F*_im_=−0.7(±0.1)*α*+79.0(±5.2), *P*<0.0001, *R*^2^=−0.57, Figure 2d), indicating that mice with narrower angles swam less than those with wider angles. These results indicate that the different outcomes of the FST for *Gabra5*^+/−^ and WT animals are conceivably not caused by a difference in emotional/behavioral state (for example, depression or helplessness), but by physical properties that also underlie the differences in *α*.

The major upward force supporting an animal in water is buoyancy, which should be reflected by the floating angles during immobility. A wider *α* corresponds to a larger part of the body being submerged, that is, the animal is less buoyant as it needs more supporting force provided by the displaced water, and vice versa. The inverse correlation between *F*_im_ and *α* may mean that buoyancy of mice could be a confounding factor in the FST.

To investigate this potential confound, we manipulated the buoyancy of mice by altering the air trapped in their fur, a key factor for keeping them afloat in water.^26, 27, 28^ Less air should be trapped in fur when reducing the surface tension of water with surfactants. Hence, soap may be used to decrease the animal's buoyancy.

A mild soap solution (1%) was gently rubbed onto the caudal areas (about 1/3 of body length) of the animals. Mice in the control group were treated similarly, but with water (0% soap solution). Soap-treated animals had significantly lower *F*_im_ (*F*_im_water_ =47.4±6.0% vs *F*_im_soap_=25.4±6.1%, *P*=0.0471, Figure 2e) and larger *α* (*α*_water_=32±1° vs *α*_soap_=55±5°, *P*<0.0001, Figure 2f), indicating that a decrease in buoyancy increases the animals' urge to swim.

To change buoyancy during the FST, we designed a customized setup for liquid exchange (Figure 1a) and devised a customized 8-min FST protocol (Figure 1b; See Materials and Methods). At the half-time point of the customized FST, we exchanged water for either water (control) or a low concentration (0.5%) soap solution. Adding soap eliminated immobility (*F*_im_epoch1_=56.2±3.6% vs *F*_im_epoch2_=0.0±0.0%, *P*<0.0001, paired two-tailed *t*-test, Figure 1c) making it impossible to measure *α* (as previously defined; See Materials and Methods). In the mild soap solution, animals assumed a vertical body posture, completely lost their ability to float and consequently were forced to swim constantly. For continuously swimming mice, it was impossible to exactly measure *α*, but as all these mice were almost straight down in the water we estimated *α* to be 90°. Therefore, adding soap solution significantly increased *α* (*α*_epoch1_=28±2° vs *α*_epoch2_=90±0°, *P*<0.0001, paired two-tailed *t*-test, Figure 1d). These results support the notion that buoyancy has confounded the results of the example experiment between *Gabra5*^+/−^ and WT animals described above. Or, in more general terms, it shows that buoyancy can affect the outcome of the FST.

In the FST, the latency to first immobility (*t*_lat_) is also considered a key measure for quantifying the state of ‘behavioral despair' of the animal.^29, 30, 31, 32, 33^ Traditionally, *t*_lat_ is the delay to the first bout of immobility lasting longer than 1 s^13, 33^ or sometimes 2 s.^30, 31^ As far as we can tell, no reason is ever given why this exact threshold of 1 or 2 s has been chosen. Hence, this threshold of 1 or 2 s is entirely arbitrary, despite the fact that it can determine the final conclusion reached (see below). To address this, we started a systematic study of the durations of immobility (*t*_im_) of all the mice used in the standard FST in water. We observed that different mice have very different distributions of *t*_im_s (Figures 3a and b): for some (for example, Figures 3a and b; open circles), most of the stops were shorter than 1 s, whereas for others (for example, Figures 3a and b; filled circles), most of the stops were much longer than the 1 or 2 s threshold. We then analyzed the very first stops of all the mice we used for standard 6-min FSTs and observed that the cumulative probability plot of the duration of the first bouts of immobility (*t*_first_im_s, Figure 3c) shows a wide distribution (*n*=57 mice), with most *t*_first_im_s longer than 1 or 2 s. The histogram of the *t*_first_im_ values follows a log-normal distribution (mean=0.5±0.1 log(s), s.d.=0.4±0.1 log(s), *R*^2^=0.65, corresponding to a mean of 3.2 ^×^ /_÷_1.3 s, Figure 3d). The wide distribution of the *t*_first_im_s may indicate that all *t*_im_s during a given FST in a single animal may be equally dispersed. Indeed, we found that in a given animal, the *t*_im_s greatly vary during the FST (Figures 3a and e). We further discovered that the cumulative probability of the *t*_im_s of a single mouse could be best fitted with the sum of two normal distributions, or in a few cases (7/68) with one normal distribution (Figure 3f; See Materials and Methods). Thus, it appears that most mice (61/68) have distinct short and long *t*_im_s. To account for the highly variable *t*_im_s, we propose that the threshold for screening the first critical immobility (*t*_c_) should be objectively determined by taking into account the means and fractional contributions of the two distributions (See Materials and Methods).

**Figure 5:**
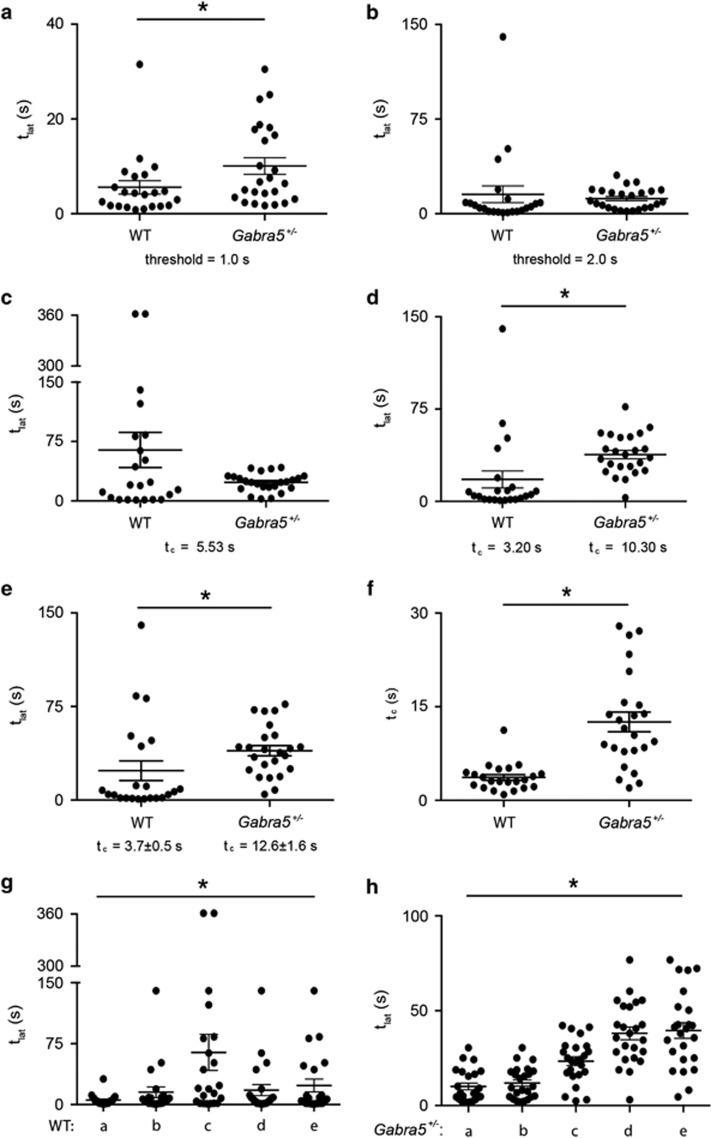
Thresholds for screening the first critical immobility. Latency to immobility (*t*_lat_) in WT (*n*=22) vs *Gabra5*^+/*−*^ (*n*=24) mice calculated with different thresholds. (**a**) Using the traditional threshold of 1 s for all the mice. (**b**) Using the traditional threshold of 2 s for all the mice. (**c**) Using a single *t*_c_ for all the mice. (**d**) Using a different *t*_c_ for each group of mice. (**e**) Using individual *t*_c_s obtained for each mouse in a given group. (**f**) Significant difference between the individual *t*_c_s of the two groups of mice. (**g** and **h**) Comparing *t*_lat_s calculated with different thresholds for WT (**g**) and *Gabra5*^+/*−*^ (**h**) mice. WT, wild-type; *t*_c_, critical threshold.

To apply and test this objective method for determining *t*_c_, we compared the effects of using different *t*_c_s to calculate *t*_lat_ of WT and *Gabra5*^+/−^ (Hets) mice in the standard FST (Figure 5, See Materials and Methods). Using the customary and arbitrary threshold of 1 s for all mice, there was a significant difference in the *t*_lat_s between the two groups (*t*_lat_WT_=5.6±1.4 s vs *t*_lat_Hets_=10.1±1.7 s, *P*=0.0231, Figure 5a). However, this significance was not there when we chose the equally arbitrary threshold of 2 s (*t*_lat_WT_=15.3±6.6 s vs *t*_lat_Hets_=11.9±1.7 s, *P*=0.0760, Figure 5b). Also, the latencies of the WT mice were significantly different when calculated with the two commonly used thresholds of 1  and 2 s (*P*=0.0313). Therefore, choosing arbitrary thresholds is utterly unreliable for calculating *t*_lat_. Next, we applied our systematic and unbiased method to define the critical thresholds (*t*_c_, See Materials and Methods). First, we used the distribution of all the *t*_im_s taken from all the mice in both groups, and got a single *t*_c_ of 5.53 s for all the animals. Using this *t*_c_, the *t*_lat_s in the two groups were not significantly different (*t*_lat_WT_=64.3±22.2 s vs *t*_lat_Hets_=23.4±2.3 s, *P*=0.9172, Figure 5c). Then, we calculated *t*_c_s for each group separately (*t*_c_WT_=3.20 s, *t*_c_Hets_=10.30 s) by using the distribution of all the *t*_im_s from each group of animals. Using these two *t*_c_s, the *t*_lat_s in the two groups became significantly different (*t*_lat_WT_=17.9±6.9 s vs *t*_lat_Hets_=38.1±3.4 s, *P*<0.0001, Figure 5d). We noticed that *t*_c_WT_ and *t*_c_Hets_ have a considerably large difference, so we then wanted to statistically test whether there is a difference between the *t*_c_s of the two groups. To do this, we used the distribution of the *t*_im_s of each mouse and determined an individual *t*_c_ for each animal. We calculated the new latencies using individual *t*_c_s, and the *t*_lat_s were significantly different between the two groups (*t*_lat_WT_=23.5±7.8 s vs *t*_lat_Hets_=39.5±4.1 s, *P*<0.0001, Figure 5e). Moreover, we did find a significant difference in the *t*_c_s between the two groups (*t*_c_individual_WT_=3.7±0.5 s vs *t*_c_individual_Hets_=12.6±1.6 s, *P*<0.0001, Figure 5f). Therefore, the new determination of *t*_c_ introduced by us can reflect important differences between the behavior of mice during the FST. Since various thresholds for both of the two groups yielded significantly different *t*_lat_ values (*P*<0.0001, Friedman test, Figures 5g and h), the critical measurement of *t*_lat_ using objectively determined *t*_c_s should be used to determine the differences in *t*_lat_.

In the current quantification of the FST, all measured criteria (for example, *F*_im_ and *t*_lat_) reflect the activities of the animals as single stationary values applied to the entire duration of the FST. But why not quantify FST behavior as it continuously changes during the test? Such a continuous quantification of behavior has been used before for the FST^34^ in rats, using kicking frequency as a readout. We devised a continuous FST behavior plot (Figure 4a) that for a given subject marks two binary states (mobile and immobile) plotted against time (resolution=0.1 s, top two plots, Figure 4a). The group behavior can be expressed by averaging the states of all animals at every time point, resulting in the probability of being mobile (*P*_mob_) as a function of time (bottom plot, Figure 4a). The plots are from the same mice shown in Figures 2e and f. Consistent with their overall activity, mice partially treated with soap had a higher *P*_mob_ throughout the test. The plots reveal that the animals started out being mobile, changing their states more frequently at first, but later becoming immobile for longer periods, resulting in a lower *P*_mob_.

More thorough analysis of the *P*_mob_ plots revealed similar synchronous behaviors in the two groups. There was an oscillation in *P*_mob_ plots in both groups regardless of the treatment (Figures 4b and c), occurring at comparable times (174.2 s and 172.5 s, Figures 4b and c) after the start of the FST, with strong behavioral alternations (controls: 0.067±0.004 Hz and part soap: 0.060±0.004 Hz, *P*=0.263), indicating that the animals change their mobile/immobile states with a cycle of 15−17 s. Notably, these analogous temporal sequences in behavior were present in both groups despite differences in their static behavioral measures (Figures 2e and f). Furthermore, the onset of this oscillatory swimming behavior must be fairly synchronous between the animals otherwise it would disappear in the averaged signal. Also, since the oscillatory behaviors start in both *P*_mob_ plots at around 173 s, all the animals appear to have a similar ‘waiting time' before starting the stop-and-go swimming behavior.

## Discussion

We addressed some pitfalls in the measurements currently used for the FST in mice. First, we uncovered buoyancy as a confounding factor in the quantification of immobility, and we propose that buoyancy of mice should be quantified to account for its impact on the interpretation of the FST. Second, we devised a systematic analysis of *t*_im_s in individual mice, defining an objective *t*_c_ for the calculation of *t*_lat_. Last, we conceived a new analysis for the temporal profile during FST behavior. Our new findings will help obtain more quantifiable results and will provide better insights into the complex behavior patterns of mice during the FST.

According to our findings, buoyancy of mice, reflected by the measured angle *α*, is a confounding factor in FST. As buoyancy partly results from air trapped in the fur, it will be affected by fur characteristics that help trap air, such as the amount of surface lipids, the length of the fur and so on.^35, 36, 37, 38^ As shown here, the animals' buoyancy could be accounted for by angle measurements and should be considered when interpreting FST results as various drugs or treatments may alter the factors responsible for trapping air in the animal's fur. We have also shown a large individual variability in the *t*_c_ that defines immobility. Therefore, we propose that *t*_c_ should henceforth be objectively defined for each animal subjected to the FST as a new variable for the test. This value should then be used for an unbiased determination of the latency to immobility (*t*_lat_) to uncover potential differences in behavior that would have previously gone unnoticed. Interestingly, we have discovered some distinct oscillatory patterns in the swimming behavior of mice during the FST. These results may indicate the presence of an invariant intrinsic biological clock that influences swimming patterns in mice, providing ground for further exciting investigations.
